# The pelvic digit “eleventh finger”

**DOI:** 10.4103/0019-5413.45332

**Published:** 2009

**Authors:** Vivek Pandey, Ajay Singh Thakur, Kiran KV Acharya, P Sripathi Rao

**Affiliations:** Department of Orthopaedics, Kasturba Medical College, Manipal, Karnataka, India

**Keywords:** Eleventh finger, pelvic digit

## Abstract

Described as asymptomatic and an incidental finding on a plain x-ray film, the “pelvic digit” is a rare congenital anomaly. A 35-year-old man is of a rare symptomatic pelvic digit that warranted surgical excision. Its importance lies in its differentiation from acquired abnormalities due to trauma such as myositis ossificans and avulsion injuries of pelvis. If this entity is kept in mind, unnecessary investigations or interventions can be avoided.

## INTRODUCTION

“Pelvic digit” is described as an asymptomatic and incidental finding on plain x-ray. It is a rare congenital anomaly, in which bone tissue develops in the soft tissue. We report a symptomatic “pelvic digit”.

## CASE REPORT

A 35-year-old man, teacher by occupation, presented with complaints of pain in the left hip of six-year duration. His pain was insidious in onset, gradually progressive, and more on walking (walking distance, 1.5 km). The pain radiated to the ipsilateral gluteal region and thigh and was relieved with rest and analgesics. There were no constitutional symptoms and there was no history of trauma. He was unable to sit cross-legged and had difficulty in climbing stairs and squatting.

**Figure 1:**
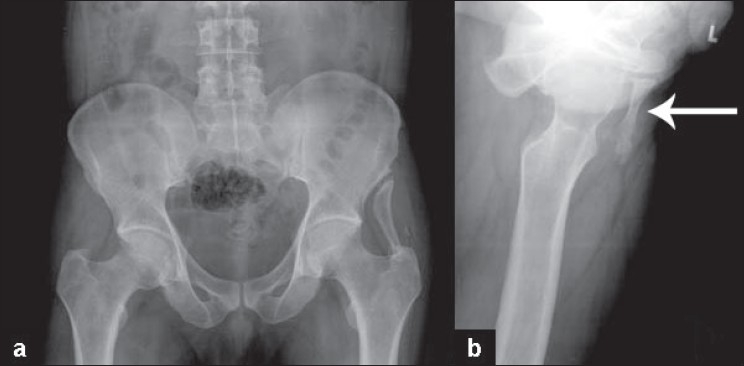
(a) Plain radiograph of the pelvis (anteroposterior view) depicting a finger-like bone projecting on the left iliac wing with pseudo-articulation with the ilium. (b) Lateral radiograph of the left hip showing a finger-like bone projecting (white arrow) on the left iliac wing.

Examination revealed a hard mass on the lateral aspect of the left hip, superior to the trochanter. The mass did not move with the trochanter. There was no tenderness in the hip joint line. He had tenderness at the upper end of the mass, near the iliac crest. His movements were as follows: flexion 0-120°, extension 0-10°, abduction 0-20°, adduction 0-10°, internal rotation 0-10°, and external rotation 0-40°. A clinical diagnosis of myositis ossificans was made. All blood investigations were within normal limits including serum calcium (8.7 mg %), phosphorus (3.3 mg %), and alkaline phosphatase (65 IU). Serum uric acid was 3.8 mg %. Plain x- ray films revealed a bony finger-like mass arising from the lateral aspect of the iliac wing, which extended up to the tip of the greater trochanter [Figure 1]. It had a clear corticomedullary differentiation and a pseudo-articulation at the iliac wing. This appearance was consistent with a pelvic digit, and hence, further investigation such as CT scan was not done. No similar history was apparent in the family. He was considered for the surgical excision of the symptomatic lesion under spinal anaesthesia. A 7 × 1.5 × 1-cm bony finger-like projection was found in the gluteus medius muscle with surrounding fibrosis. There was a fibrous joint noted at the iliac wing. The bony “finger” extended from the iliac wing to the tip of the trochanter. The mass was excised along with its periosteum. It was cut open to find a clear bone-like corticomedullary differentiation. Hip movements improved postoperatively, and the local pain was relieved.

## DISCUSSION

The pelvic digit was first reported by Sullivan and Cornwell in 1974, the lesion can be located at any level of the pelvic bones, lower ribs, or even in the anterior abdominal wall.^1^–^3^ It may or may not pseudo-articulate with the axial skeleton.^4^ In this case, the patient's radiograph typically showed a ribor digit-like bony structure with a clear cortex and medulla and with a pseudo-articulation. Although described as a benign entity, which is usually discovered incidentally, this was symptomatic, causing pain and restriction of movements.^5^ To the best of our knowledge, this is first case report of a symptomatic pelvic digit.^2^,^3^,^6^ Radiological differentiation from heterotopic ossification (post-traumatic myositis ossificans) and avulsion fractures of the pelvis can be made because of the typical appearance of the pelvic digit and the absence of a history of trauma.^7^ The origin of the pelvic digit is not yet established. The most likely theory is that the anomaly arises in the mesenchymal stage of bone growth within the first six weeks of embryogenesis. Normally, the independent cartilaginous costal primordium of the first coccygeal vertebra fuses with the vertebral column. If the fusion does not take place, the cartilaginous centre may develop independently, forming a rudimentary “rib”.^3^ The segmentation of these cartilaginous centers might cause the digit-like appearance hence also called as an extra eleventh finger.^8^ Other theories suggest its origin from the pleuripotentiality of mesenchymal stem cell tissues.^2^

## CONCLUSION

The radiographic entity of pelvic rib or digit, described earlier as an incidental finding, can be symptomatic, causing pain and restriction of movements and requiring surgical excision. Recognition of this entity is important, as it is usually benign and if asymptomatic, it is best left alone.^8^
